# Beyond Survival: Parents’ Experiences and Healthcare Professionals’ Perspectives on Family-Centred Care for Children with Congenital and Acquired Heart Disease: A Qualitative Study

**DOI:** 10.3390/healthcare14183018

**Published:** 2026-09-15

**Authors:** Esma Nur Ünal, Umut Berk Mercan, Ebru Aypar, Sıddika Songül Yalçın

**Affiliations:** 1 Department of Paediatrics, Faculty of Medicine, Hacettepe University, 06230 Ankara, Türkiye; 2 Division of Paediatric Cardiology, Department of Paediatrics, Faculty of Medicine, Hacettepe University, 06230 Ankara, Türkiye; berk955@gmail.com (U.B.M.); ebruaypar@gmail.com (E.A.); 3 Unit of Social Paediatrics, Department of Paediatrics, Faculty of Medicine, Hacettepe University, 06230 Ankara, Türkiye

**Keywords:** congenital heart disease, acquired heart disease, qualitative study, caregiver, parents’ experiences, healthcare professionals’ perspective, cardiac care, healthcare need, psychosocial need

## Abstract

**Highlights:**

**What are the main findings?**

- Parents and healthcare professionals viewed childhood congenital and acquired heart disease as a long-term developmental and family journey extending beyond diagnosis, treatment, and survival.
- Family adaptation evolved through psychological adjustment, caregiver empowerment, communication, and increasing caregiving expertise, while healthcare professionals identified similar unmet needs across the care trajectory.

**What are the implications of the main findings?**

- Family-centred paediatric cardiac care should integrate developmental surveillance, psychosocial support, caregiver education, and multidisciplinary collaboration into routine long-term follow-up.
- Improving continuity of care and strengthening partnerships between families and healthcare professionals may enhance developmental outcomes, family well-being, and lifelong quality of care for children with heart disease.

**Abstract:**

**Background**: As survival increases among children with congenital and acquired heart disease (HD), understanding how families adapt to long-term care and family needs has become increasingly important. This study explored parents’ experiences and healthcare professionals’ perspectives to identify unmet needs and inform family-centred paediatric cardiac care. **Methods**: A qualitative study informed by a constructivist perspective was conducted. In-depth semi-structured interviews were conducted with 39 parents (32 mothers and 7 fathers) of young children with congenital or acquired HD recruited from a single tertiary paediatric cardiology centre, and six healthcare professionals recruited from three different tertiary centres. Data were analysed using inductive reflexive thematic analysis following Braun and Clarke’s six-phase approach. Bronfenbrenner’s Ecological Systems Theory was subsequently used as a sensitising framework to support interpretation of the findings. **Results**: Six interrelated themes emerged: (1) childhood HD as a long-term developmental journey beyond survival; (2) the impact on everyday life and family participation; (3) parents’ progressive psychological adaptation; (4) the central role of communication and continuity of care; (5) parents’ transformation into expert caregivers; and (6) healthcare professionals’ support for integrated family-centred care. Parents described caregiving as an evolving process characterised by uncertainty, emotional adaptation, increasing caregiving competence, and changing family roles. Healthcare professionals similarly highlighted the need to address developmental, psychosocial, nutritional, and educational needs alongside specialist cardiac management. **Conclusions**: The findings suggest that childhood HD may be understood not only as a chronic medical condition but also as a long-term developmental and family experience. Parents’ and healthcare professionals’ complementary perspectives highlight the need for integrated family-centred paediatric cardiac care that combines developmental surveillance, caregiver empowerment, multidisciplinary collaboration, and continuity of care throughout the disease trajectory.

## 1. Introduction

Congenital and acquired heart diseases (HDs) are among the leading chronic conditions affecting children worldwide and remain important causes of childhood morbidity despite substantial improvements in survival. Congenital HD, the most common congenital anomaly, affects approximately 9 per 1000 live births globally [1,2]. Advances in prenatal diagnosis, perioperative care, interventional cardiology, and cardiac surgery have dramatically improved outcomes, allowing more than 95% of children with congenital HD to survive into adulthood. As a result, the focus of paediatric cardiac care has gradually shifted beyond survival toward optimising long-term health, development, and quality of life [3,4,5,6].

Early childhood represents a critical period for physical, cognitive, emotional, and social development. According to the Nurturing Care Framework, optimal child development depends on the interaction of five essential domains: good health, adequate nutrition, responsive caregiving, opportunities for early learning, and safety and security [7]. Although survival has improved substantially, children with HD remain vulnerable to developmental delays and impairments across multiple domains, including neurodevelopment, cognition, motor function, behaviour, and psychosocial well-being [6,8]. Disease-related factors, repeated hospitalisations, surgical interventions, and prolonged medical follow-up may interfere with normal developmental trajectories, increasing the risk of feeding difficulties, poor growth, sleep disturbances, reduced physical activity, and limited social participation [9,10]. Consequently, comprehensive care for children with HD should extend beyond medical treatment to include developmental monitoring and family-centred support.

The impact of paediatric HD extends well beyond the affected child. Parents frequently experience persistent uncertainty regarding prognosis, repeated exposure to stressful medical events, and responsibility for complex caregiving tasks. Managing medical treatments while supporting the child’s developmental, emotional, and social needs places a substantial psychological and practical burden on families. Previous studies have consistently demonstrated higher levels of parental stress, anxiety, depression, and caregiver burden among families of children with chronic HD compared with families of healthy children [11,12]. Inadequate communication, insufficient information, and fragmented psychosocial support may further intensify these challenges and influence both parental well-being and child outcomes [13,14,15].

Although increasing attention has been given to neurodevelopmental outcomes and family-centred care in paediatric cardiology [6,8,11], relatively little is known about how families experience the entire care journey within the healthcare system, particularly from diagnosis through long-term follow-up [12,13]. Most previous studies have focused on specific clinical stages or discrete aspects of care, providing limited insight into parents’ lived experiences and their interactions with healthcare services across the disease trajectory. Although a small number of qualitative studies have incorporated the perspectives of both parents and healthcare professionals, these have largely focused on specific domains. For example, Woolf-King et al. [16] focused primarily on parental mental health and the integration of psychological support into care for families of children with critical congenital heart disease; Saxena et al. [17] examined healthy active lifestyles, including physical activity and related barriers and supports, among children with complex heart disease, families, and professionals; and Nemes et al. [18] explored parents’ and clinicians’ experiences of growing up with congenital heart disease, including communication, psychosocial challenges, and physical activity. However, these studies were restricted to congenital or complex heart disease populations and/or specific domains of care. Consequently, evidence integrating parent and healthcare professional perspectives across the broader clinical, developmental, psychosocial, and healthcare-system trajectory remains limited, particularly in samples including children with both congenital and acquired HD.

Therefore, this qualitative study aimed to explore parents’ experiences throughout their children’s disease trajectories, focusing on children’s growth, development, everyday functioning, and healthcare experiences. Specifically, the study addressed three research questions: (1) How do parents experience and navigate the clinical, developmental, and emotional trajectories associated with their children’s HD? (2) How do families adapt their everyday lives and mobilize coping resources across the disease trajectory? and (3) How do healthcare professionals perceive the barriers to and requirements for integrated family-centred care? By integrating the perspectives of parents and healthcare professionals, this study aimed to identify unmet healthcare and psychosocial needs and to inform approaches to family-centred, multidisciplinary care for children with HD and their families.

## 2. Materials and Methods

### 2.1. Study Design and Reporting

This qualitative study was informed by a constructivist perspective, recognising that participants construct meaning through their individual experiences and interactions within social and healthcare contexts. Semi-structured, in-depth interviews were therefore used to explore parents’ caregiving experiences, their perceptions of their children’s developmental and everyday needs, interactions with healthcare services, and unmet support needs, as well as healthcare professionals’ perspectives on family-centred paediatric cardiac care. The study was reported in accordance with the Consolidated Criteria for Reporting Qualitative Research (COREQ) 32-item checklist to support methodological transparency and comprehensive reporting [19,20].

### 2.2. Research Setting

The parent component of the study was conducted at Hacettepe University İhsan Doğramacı Children’s Hospital, one of the largest tertiary paediatric referral centres in Türkiye, where children with congenital and acquired HDs receive comprehensive diagnostic, medical, and surgical care. Healthcare professionals were recruited from three tertiary healthcare institutions, all providing specialist paediatric cardiac care.

Parent interviews were conducted in a private consultation room within the Social Paediatrics Outpatient Clinic to ensure confidentiality, privacy, and a comfortable interview environment [20].

Healthcare professionals were interviewed individually in quiet rooms within their respective departments outside routine clinical hours to minimise interruptions to patient care and facilitate open discussion.

Data collection was undertaken between September 2025 and March 2026.

### 2.3. Participants, Sampling and Recruitment

The study population consisted of two participant groups: (1) parents of children aged 0–8 years diagnosed with congenital or acquired HD and receiving follow-up care at Hacettepe University İhsan Doğramacı Children’s Hospital, and (2) healthcare professionals directly involved in the diagnosis, treatment, follow-up, or nursing care of paediatric HD.

Participants were selected using purposive sampling to recruit individuals with direct and information-rich experience relevant to the research questions. To capture a broad range of perspectives while preserving the depth of individual accounts, maximum variation sampling was used, informed by Patton’s information-rich case strategy [21]. Rather than achieving statistical representativeness, this approach sought to capture common patterns across diverse clinical and family contexts.

Variation among parent participants was achieved by considering the child’s diagnosis (congenital or acquired HD), treatment history (with or without previous cardiac surgery), presence of additional chronic conditions, and parental role (mother or father). Fathers were intentionally recruited to broaden the range of parental caregiving perspectives represented in the dataset beyond maternal accounts alone. Congenital and acquired HDs were included to capture variation in clinical trajectories and caregiving contexts rather than to enable comparisons between diagnostic groups. The study focused on cross-cutting parental experiences and family needs associated with caring for a child with HD, while attention was also paid during the analysis to potentially divergent experiences related to different disease trajectories.

Variation among healthcare professionals was ensured by including different professional disciplines involved in paediatric cardiac care, including paediatric cardiologists, cardiovascular surgeons, and specialised nurses. This multidisciplinary sampling enabled exploration of the healthcare process from complementary professional perspectives.

Eligible parents were approached during routine outpatient follow-up visits by the researcher after completion of their child’s clinical evaluation. Healthcare professionals were invited individually according to their clinical roles and experience in paediatric cardiac care. All eligible participants received verbal and written information regarding the study and provided written informed consent before participation.

Recruitment was guided by the study aims, purposive maximum variation sampling strategy, and the information richness and diversity of participants’ accounts. A total of 39 parents and 6 healthcare professionals participated in the study. The healthcare professional sample was intended to provide a focused, complementary perspective across different disciplines and tertiary-care settings rather than to achieve independent thematic saturation or professional representativeness.

The parent sample included:

- Parents of children with congenital HD without previous cardiac surgery (n = 18);
- Parents of children with congenital HD who had undergone cardiac surgery (n = 15);
- Parents of children with acquired HD (n = 6);
- Parents of children with additional chronic comorbidities (n = 8). Because some children had both HD and accompanying chronic conditions, participants in the latter category overlapped with other diagnostic groups.

At least six participants were recruited from each clinical subgroup, and at least one father was included in each group to maximise diversity of parental experiences. The acquired HD subgroup was included to broaden the range of disease trajectories represented in the dataset and was not intended to support a separate condition-specific comparison with congenital HD.

The inclusion and exclusion criteria for both participant groups are presented in Table 1.

### 2.4. Data Collection

Data were collected through individual semi-structured, face-to-face interviews, which enabled participants to describe their experiences in their own words while allowing the interviewer to explore issues arising during the conversation. Separate interview guides were developed for parents and healthcare professionals following a review of the literature and discussions among the multidisciplinary research team. The interview guides included open-ended questions exploring the diagnostic process, caregiving experiences, children’s growth and development, daily life, healthcare experiences, communication with healthcare professionals, psychosocial impacts, support systems, and unmet healthcare needs (Supplementary Materials S1 and S2). The guides were pilot-tested with two parents to assess clarity, comprehensibility, and flow; no major modifications were required, and the pilot interviews were not included in the final analysis.

Because the interviews explored experiences across the child’s disease trajectory, parents were asked to reflect on clinical events occurring at different stages, including diagnosis, treatment, and, where applicable, surgery. Given the children’s broad age range (0–8 years) and variation in the timing of diagnosis and treatment, the time elapsed between these clinical events and the interview varied across participants, ranging from relatively recent experiences to events that had occurred several years earlier.

All interviews were conducted by a social paediatrician (SSY) with extensive experience in qualitative research, together with a paediatric resident (ENÜ) who had received training in qualitative interviewing. Neither interviewer had a prior personal or clinical relationship with the participants before study commencement, and neither was a primary physician for any of the participants. Before each interview, the interviewers introduced themselves and their roles in the study, and a brief introductory conversation was used to establish rapport and facilitate an open, non-judgmental dialogue. Reflexive field notes documenting contextual observations, interviewer reflections, and emerging analytical insights were recorded immediately after each interview. Interviews lasted approximately 35–75 min and were audio-recorded with participants’ permission. Audio recordings were transcribed verbatim shortly after each interview and checked against the recordings to ensure transcription accuracy. Identifiable information was removed during transcription to maintain participant confidentiality.

### 2.5. Research Team and Reflexivity

The multidisciplinary research team consisted of paediatricians with expertise in social paediatrics, child health, paediatric cardiology, qualitative research, and health services research. All interviews were conducted by the same two interviewers—a social paediatrician experienced in qualitative research (SSY) and a paediatric resident trained in qualitative interviewing (ENÜ)—both of whom were subsequently involved in coding and initial theme development. The involvement of the same two researchers throughout data collection and initial analysis supported continuity between interviewing, contextual understanding, and engagement with the data. Neither interviewer was involved in the participants’ routine clinical care or served as their primary physician. At the same time, the researchers recognized that their clinical backgrounds could shape the issues explored during interviews, the use of follow-up questions, coding, and the interpretation of participants’ accounts. A common semi-structured interview guide (Supplementary Materials S1 and S2) was used by both interviewers to ensure consistency in the core topics explored, while allowing participants to elaborate on issues they considered important. The interviewers also discussed their interviewing practices during data collection to reflect on potential differences in probing and questioning style.

Recognising that researchers inevitably influence qualitative inquiry, reflexivity was integrated throughout the study. Reflexive field notes and analytical memos were recorded immediately after each interview to document contextual observations, interviewer reflections, emerging interpretations, and the researchers’ assumptions and positioning in relation to the data. These records were revisited throughout the analytic process to support critical self-reflection and enhance analytic transparency. Particular attention was paid to how the research team’s clinical experience and prior assumptions about family-centred care might shape the interpretation of participants’ accounts. Rather than attempting to eliminate or fully bracket these assumptions, the researchers sought to make them explicit and critically examine their influence throughout the analytic process.

Throughout coding and initial theme development, SSY and ENÜ discussed their coding decisions and emerging interpretations and jointly refined the developing analysis through engagement with the data. Where their initial interpretations differed, these differences were explored through discussion and re-engagement with the relevant data to reach a shared analytical interpretation. This collaborative process was used to broaden and deepen interpretation rather than to assess inter-coder reliability or establish coding agreement as a measure of analytic validity. Developing themes were subsequently discussed within the multidisciplinary research team to question assumptions, consider alternative interpretations, and refine their conceptual boundaries and meanings. These team discussions were similarly used to enrich interpretation rather than to establish a single objectively correct coding framework.

### 2.6. Data Analysis

Data were analysed using inductive reflexive thematic analysis, following the six-phase approach described by Braun and Clarke [22]. This approach was selected because it facilitates an in-depth exploration of participants’ accounts while identifying patterns of shared meaning across the dataset without imposing predefined categories.

Data analysis proceeded concurrently with data collection, allowing emerging concepts to inform subsequent interviews while preserving the overall structure of the interview guide. As data collection progressed, preliminary engagement with the interview material was used to assess the breadth and depth of the developing dataset and the contribution of subsequent interviews to the research questions. Consistent with reflexive thematic analysis, thematic saturation was not treated as a fixed or objectively verifiable endpoint, and no numerical threshold for code saturation was used as a stopping criterion. Rather, data collection was concluded when the dataset was considered sufficiently rich and diverse to support an in-depth interpretation of the experiences relevant to the study aims. The analysis involved the following iterative stages:

**Familiarization with the data.** Both interviewers (SSY and ENÜ) repeatedly read all transcripts while reviewing field notes and reflexive memos to develop an in-depth understanding of participants’ narratives.

**Initial coding.** Transcripts were imported into MAXQDA Analytics Pro 24 (VERBI Software, Berlin, Germany). SSY and ENÜ were both actively involved in inductive coding of the dataset. Coding decisions and developing interpretations were discussed collaboratively and refined through joint engagement with the data. This process was used to explore different interpretations rather than to assess inter-coder reliability or establish coding agreement as a measure of analytic validity.

**Generating themes.** The researchers iteratively examined the codes and developing interpretations to generate candidate themes representing broader patterns of shared meaning across the dataset. Candidate themes were subsequently discussed within the multidisciplinary research team, with differing perspectives used to question assumptions, consider alternative interpretations, and refine theme development.

**Reviewing and refining themes.** Candidate themes were reviewed against the complete dataset, with attention to internal coherence, clear distinctions between themes, and consistency with participants’ narratives. During this process, attention was also paid to both shared and potentially divergent patterns across congenital and acquired HD trajectories. No sufficiently developed condition-specific patterns were identified within this dataset to warrant separate thematic structures for congenital and acquired HD. Themes were modified, merged, or separated through iterative discussion among the research team.

**Defining and naming themes.** Each final theme was clearly defined to reflect its central meaning and its relationship with other themes. Particular attention was paid to preserving the complexity of participants’ experiences while ensuring conceptual clarity.

**Interpretation.** Although themes were generated inductively from the data, Bronfenbrenner’s Ecological Systems Theory was subsequently used as a sensitising theoretical framework during interpretation [23]. This framework facilitated understanding of how individual experiences were shaped by interactions among child-, family-, healthcare-, organisational-, and health system-level factors, without constraining the initial coding process.

Throughout the analysis, patterns of meaning were examined iteratively within and across interviews, with attention to both shared and divergent perspectives. Parent and healthcare professional accounts were initially analysed in relation to their respective research questions and were subsequently considered together during interpretation to identify complementary perspectives on family and healthcare-system experiences.

### 2.7. Trustworthiness

The trustworthiness of the study was established according to the four criteria proposed: credibility, dependability, confirmability, and transferability [24].

**Credibility** was supported through purposive maximum variation sampling, iterative engagement with the dataset, reflexive discussion within the multidisciplinary research team, and extensive use of verbatim quotations to illustrate participants’ perspectives. The inclusion of parents and healthcare professionals provided complementary perspectives on family-centred cardiac care rather than serving as a form of confirmatory validation.

**Dependability** was ensured by maintaining a transparent audit trail documenting methodological decisions, interview procedures, coding development, theme refinement, and analytical discussions. The use of a semi-structured interview guide also promoted consistency across interviews while allowing sufficient flexibility to explore participants’ individual experiences.

**Confirmability** was supported through reflexive field notes, analytical memos, and a transparent audit trail documenting coding decisions and theme development. Team discussions were used to critically examine interpretations while acknowledging the active role of researchers in knowledge production.

**Transferability** was supported by providing detailed descriptions of the study context, participant characteristics, recruitment strategy, healthcare setting, and analytical procedures. Maximum variation sampling enabled the inclusion of participants with diverse clinical and caregiving experiences, thereby allowing readers to assess the applicability of the findings to similar healthcare contexts.

## 3. Results

### 3.1. Participant Characteristics

Forty-five participants were interviewed, including 39 parents (32 mothers and 7 fathers) of children with HD (Table 2) and six healthcare professionals representing paediatric cardiology, cardiovascular surgery, intensive care nursing, and outpatient care (Table 3). Parent participants represented children with a broad spectrum of congenital and acquired HD, providing diverse perspectives across different stages of diagnosis, treatment, surgery, and long-term follow-up.

### 3.2. Overview of the Findings

Reflexive thematic analysis identified six overarching themes and twenty-five subthemes (Table 4). Although themes are presented separately for reporting purposes, they represent interconnected experiences extending across the child’s clinical journey, family adaptation, healthcare interactions, and long-term management. Themes 1–5 were derived from parent interviews, whereas Theme 6 was derived from healthcare professional interviews. Although some concerns overlapped across the two participant groups, particularly regarding communication, continuity of care, developmental and psychosocial needs, and coordination of services, these accounts were interpreted as complementary perspectives rather than as confirmatory evidence.

### 3.3. Theme 1. Living Through the Clinical Journey: From Diagnosis to Long-Term Care

Children’s clinical journeys were characterised by a dynamic process extending from the first recognition of symptoms to long-term follow-up after diagnosis and treatment. Although disease severity and clinical trajectories varied considerably across participants, parents described experiences marked by uncertainty, repeated healthcare encounters, major treatment decisions, and gradual adaptation over time. Four interrelated subthemes captured this journey: (1) receiving the diagnosis; (2) navigating treatment and surgical care; (3) growth, nutrition, and developmental trajectories; and (4) recovery beyond survival (Table 4).

#### 3.3.1. Receiving the Diagnosis: Uncertainty, Delays, and Referral Pathways

Families experienced remarkably disparate pathways to a definitive diagnosis. Although some children were diagnosed prenatally or shortly after birth, others faced an extended clinical course marked by repeated consultations, sequential referrals, and occasional misdiagnoses. The age at detection spanned from infancy to late childhood, often triggered by the evaluation of persistent symptoms like heart murmurs, recurrent respiratory infections, fever, or edema, as well as incidental findings. Reaching a specialised tertiary paediatric cardiology centre frequently required navigating several intermediary healthcare facilities. These systemic delays—largely attributed to regional deficits in paediatric cardiology expertise, inadequate diagnostic resources, and heterogeneous clinical assessments—compounded parental frustration, uncertainty, and emotional burnout during the search for answers.

“*One doctor said it was teething, another thought it was hand-foot-and-mouth disease. When my child continued to deteriorate, I took her to another hospital, and only then was the correct diagnosis made.*”

(P35, Mother, Acquired HD [KD], Duration for diagnosis: 5 days)

#### 3.3.2. Navigating Treatment and Surgical Care

Following diagnosis, families entered a prolonged treatment trajectory involving medical management, repeated follow-up visits, and, for many children, cardiac surgery. Surgical intervention emerged as a major turning point rather than the endpoint of care. Parents described surgery simultaneously as a life-saving opportunity and one of the most emotionally challenging experiences of the disease trajectory.

Children underwent a broad range of interventions, including catheter-based procedures, corrective cardiac operations, staged surgeries, and rhythm interventions such as catheter ablation. Decisions regarding surgery were influenced not only by immediate clinical indications but also by concerns regarding growth failure, declining cardiac function, and prevention of future complications.

Despite high levels of preoperative anxiety, parents consistently reported noticeable improvements following successful interventions. Recovery was reflected not only in improved cardiac status but also in enhanced overall health status, reduced respiratory symptoms, better sleep, increased appetite, and greater participation in everyday activities.

“*After the procedure, she finally told me she could run with her friends without being afraid that her heart would race.*”

(P39, Mother, HD [SVT])

Nevertheless, recovery was not always straightforward. Some children experienced prolonged intensive care admissions, multiple operations, or treatment-related complications, including temporary developmental regression, vocal cord paralysis, or delayed motor recovery. Parents described these complications as additional challenges requiring long-term adaptation rather than isolated postoperative events.

#### 3.3.3. Growth, Nutrition, and Developmental Trajectories

Parents consistently viewed their children’s growth and developmental progress as key indicators of health beyond cardiac outcomes. Feeding difficulties, poor weight gain, delayed motor development, and speech problems were frequently described, particularly among children with more complex cardiac disease or prolonged hospitalisation.

Breastfeeding experiences varied considerably. While many children were successfully breastfed, others required infant formula because of prolonged hospitalisation, sucking difficulties, intensive care admission, poor weight gain or maternal concerns regarding milk supply. Complementary feeding generally began around six months of age, although medical complications and feeding refusal delayed this transition in several children.

Parents often evaluated treatment success by observing improvements in appetite, weight gain, physical growth, and developmental milestones rather than solely by medical indicators. Although many children eventually achieved age-appropriate developmental milestones, others experienced delayed walking, speech difficulties, or temporary regression following prolonged hospitalisation.

One parent challenged common societal assumptions regarding poor growth in congenital HD by stating:

“*People always said that children with HD grow slowly, but my baby was actually quite chubby.*”

(P2, Mother, congenital HD [VSD])

These narratives illustrated that growth and development represented important markers through which families interpreted both disease severity and recovery.

#### 3.3.4. Recovery Beyond Survival

Parents emphasized that successful treatment extended far beyond survival or surgical success. Recovery was understood as a gradual process of regaining normal childhood activities, improving physical functioning, and restoring family life. Clinical improvement was frequently accompanied by better sleep quality, increased physical activity, enhanced social participation, and reduced parental anxiety.

However, several families explained that recovery remained incomplete despite successful cardiac treatment. Children who experienced repeated hospitalisations or complex surgical procedures continued to face developmental, psychological, or functional challenges requiring ongoing multidisciplinary follow-up. Consequently, parents viewed long-term care as a continuous process rather than the conclusion of treatment.

Across interviews, families consistently described the transition from diagnosis to long-term management as a journey requiring repeated adaptation. Although medical interventions substantially improved children’s health, parents regarded optimal recovery as encompassing physical growth, developmental progress, emotional well-being, and participation in everyday life.

### 3.4. Theme 2. Living with HD in Everyday Life

Beyond medical treatment, parents described congenital and acquired HD as conditions that continuously shaped children’s everyday routines, lifestyle behaviours, and participation in family life. Daily care extended far beyond medication administration, encompassing feeding practices, sleep routines, physical activity, preventive health behaviours, media use, and environmental adaptations. Rather than representing isolated caregiving activities, these practices reflected families’ ongoing efforts to balance medical protection with the child’s developmental needs. Four interrelated subthemes emerged (Table 4).

#### 3.4.1. Establishing Daily Routines Around a Chronic Condition

Parents described daily life as becoming highly structured around their child’s health needs. Medication schedules, medical appointments, nutritional monitoring, and observation of symptoms became integrated into everyday family routines. As children grew older, many families gradually developed individualized strategies that allowed disease management to coexist with ordinary childhood activities. Several parents emphasized that living with HD eventually became “part of normal life.” Nevertheless, maintaining this sense of normality required continuous monitoring and adjustment according to the child’s health status.

#### 3.4.2. Nutrition, Sleep, and Physical Activity as Indicators of Well-Being

Nutrition, sleep quality, and physical activity were consistently viewed as practical indicators of children’s overall health and recovery. Parents closely monitored appetite, weight gain, sleeping patterns, and exercise tolerance, interpreting improvements in these areas as signs of successful disease management rather than solely relying on clinical examinations.

Sleep patterns varied considerably between children. While many children slept through the night without difficulty, others experienced frequent awakenings related to thirst, feeding, or previous hospitalisation experiences. Parents also described changes in sleep behaviours following surgery, with many reporting substantial improvements once respiratory symptoms or cardiac instability resolved.

Physical activity represented another important aspect of daily life. Although protecting the child from excessive physical strain remained a common parental concern, many families consciously avoided unnecessary restrictions. Healthcare professionals frequently encouraged disease-appropriate physical activity, and parents recognized that excessive limitations could negatively affect children’s psychological well-being and social participation.

“*She can run, play, and climb stairs without difficulty. In fact, the doctors encourage her to stay physically active.*”

(P6, Mother, Congenital HD [BAV])

Some parents also observed that, over time, children learned to recognize their own physical limits and adjusted their activities accordingly.

#### 3.4.3. Preventive Health Practices and Environmental Adaptations

Families adopted a wide range of preventive strategies aimed at reducing health risks associated with chronic HD. These included adherence to routine immunizations, additional recommended vaccines, vitamin and iron supplementation, infection prevention measures, and modifications of the home environment.

Parents frequently described heightened attention to hygiene practices, avoidance of crowded environments during periods of increased infection risk, and strict household smoking rules. Even in families where parents smoked, smoking was generally restricted to outdoor areas in order to minimize children’s exposure to tobacco smoke.

Although these measures were considered essential for protecting children’s health, some families acknowledged that prolonged infection avoidance occasionally reduced opportunities for social interaction outside the home.

#### 3.4.4. Participation in Everyday Childhood

Families continuously negotiated the balance between protecting their child and supporting participation in ordinary childhood experiences. Parents sought to preserve normal routines whenever possible while remaining attentive to the child’s physical limitations. Hospitalisations, surgical procedures, and recovery periods frequently interrupted regular family life, requiring adaptations in schooling, play, and recreational activities.

Media use emerged as one of the adaptive strategies used during prolonged hospitalisations and recovery periods. Parents described televisions, tablets, and mobile phones not only as recreational devices but also as practical tools for facilitating feeding, reducing procedural distress, maintaining bed rest, or distracting children during lengthy treatments.

“*My child had never used a tablet before. During prolonged intravenous treatment, it became the only way to keep her calm in bed.*”

(P35 Mother, Acquired HD [KD])

Despite these adaptations, many parents consciously attempted to limit screen exposure and encourage age-appropriate social interaction whenever health conditions allowed.

Overall, everyday life with HD was characterised by continuous adjustment rather than constant restriction. Families sought to integrate medical care into ordinary childhood routines while preserving opportunities for development, play, independence, and participation. These findings suggest that successful long-term management extends beyond clinical stability and includes supporting children’s ability to engage in everyday life as fully as possible.

### 3.5. Theme 3. Parents’ Psychological Responses and Emotional Processing Across the Disease Trajectory

While practical coping strategies are addressed separately, this theme captures the internal, longitudinal emotional processing of parents as they navigate the disease trajectory. Reflexive thematic analysis revealed distinct differences in the initial phases of the parents’ journeys: parents of children with acquired heart disease often described a sudden, unexpected onset that severely disrupted family equilibrium, whereas congenital cases were typically identified early or prenatally. However, despite these divergent diagnostic entry points, the subsequent chronic caregiving demands, parental hypervigilance, and challenges in healthcare system navigation were profoundly similar across both groups. Parents described their experiences as an evolving emotional journey that extended well beyond the initial diagnosis. Emotional responses changed over time, moving from shock and uncertainty during diagnosis to adaptation, hope, persistent vigilance, and concerns about the future. Rather than occurring as isolated reactions, these emotions continuously interacted with children’s clinical progress, treatment experiences, and everyday family life. Five interconnected subthemes described this trajectory (Table 4).

#### 3.5.1. Confronting the Diagnosis: Shock, Fear, and Meaning-Making

The moment of diagnosis represented one of the most emotionally intense periods described by parents. Regardless of whether the diagnosis occurred prenatally or after birth, families frequently recalled feelings of shock, fear, helplessness, sadness, and an overwhelming fear of losing their child. Many described the diagnosis as a life-changing event that immediately altered their expectations of parenthood.

After the diagnosis, some parents attributed blame to themselves or others. One father (P36, Acquired HD [ARF]) blamed himself for not seeking earlier treatment for his child’s throat infection. Similarly, one mother (P37, Acquired HD [KD]) expressed deep regret, stating, “*I wish I had gone to this doctor earlier,*” while another (P2, Congenital HD [VSD]) questioned whether her physical exhaustion during pregnancy caused the anomaly. Blame was also projected outwardly; for instance, a mother (P22, Congenital HD [DCM]), navigating the diagnosis while her husband was incarcerated, blamed him directly, stating, “*If you had looked after your children, this wouldn’t have happened.*”

Several parents attempted to make sense of the diagnosis by searching for possible explanations. Some questioned events during pregnancy, previous healthcare decisions, or environmental factors, whereas others interpreted the diagnosis through religious beliefs and acceptance. Rather than representing opposing coping styles, both active meaning-making and spiritual acceptance appeared to coexist throughout the adjustment process.

“*When we learned about the diagnosis, it felt as though my whole world had collapsed.*”

(P9, Mother, Congenital HD [TGA])

Others expressed acceptance through faith:

“*Whatever God has written for us, we accepted it wholeheartedly.*”

(P3, Mother, Congenital HD [TOF])

These narratives illustrated how parents simultaneously experienced profound emotional distress while searching for ways to regain a sense of control over an uncertain situation.

#### 3.5.2. Living with Uncertainty Throughout the Disease Course

Although emotional distress was greatest at diagnosis, uncertainty remained a persistent feature throughout the child’s illness. Parents described waiting for follow-up examinations, monitoring surgical decisions, and anticipating possible complications as emotionally exhausting experiences. Many explained that uncertainty was psychologically more difficult than receiving unfavourable medical information because it prolonged feelings of helplessness and prevented families from planning for the future. Even after successful treatment, parents remained vigilant, frequently monitoring their child’s breathing, activity level, or physical condition.

One mother described this prolonged hypervigilance:

“*Even after everything improved, I still woke up during the night just to check whether she was breathing.*”

(P35, Mother, Acquired HD [KD])

Several parents reported that these persistent worries eventually required professional psychological support.

#### 3.5.3. Rebuilding Hope Following Treatment

Despite the emotional burden associated with surgery, successful treatment frequently transformed parents’ perceptions of the future. Surgery was commonly described not only as a medical intervention but also as a turning point that restored hope and renewed expectations for childhood development. Parents associated improvements in physical health with emotional recovery. Observing their children eating better, becoming more active, sleeping peacefully, or participating in everyday activities strengthened confidence in the future and reduced immediate anxiety. Some parents also described feelings of gratitude after witnessing children with more severe conditions during hospitalisation, leading them to reinterpret their own experiences from a broader perspective. Nevertheless, hope did not completely eliminate fear. Instead, parents described learning to live with both optimism and ongoing uncertainty.

#### 3.5.4. Reshaping Family Life and Parenting Roles

The child’s illness fundamentally changed family dynamics. Mothers generally assumed primary responsibility for medical care, daily monitoring, and communication with healthcare professionals, whereas fathers frequently described their role as providing emotional stability, practical assistance, and opportunities for play and social interaction.

Parents also described how family routines gradually became organized around the child’s health needs. Daily schedules, employment decisions, family activities, and sibling relationships were continuously adjusted according to treatment requirements and concerns about infection or physical safety. Although many families acknowledged becoming increasingly protective over time, several consciously attempted to avoid creating a “sick child” identity. These parents emphasized treating their children similarly to healthy siblings in order to support independence and social inclusion.

“*My child is just like every other child. We never wanted her to grow up believing she was different.*”

(P39, Mother, HD [SVT])

#### 3.5.5. Looking Toward an Uncertain Future

Long-term uncertainty remained one of the defining characteristics of parents’ experiences. Families recognized that HD represented a chronic condition requiring lifelong monitoring rather than a problem completely resolved through surgery or medical treatment.

Parents expressed concerns regarding future operations, educational participation, employment opportunities, independent adulthood, and the possibility of unforeseen complications. These concerns were often intensified by the unpredictable nature of disease progression.

“*The uncertainty is what frightens me most. We know we have to wait, but we never really know what comes next.*”

(P31, Mother, Congenital HD [ASD])

Despite these concerns, many parents described reaching a stage of psychological adaptation in which uncertainty became integrated into everyday life rather than dominating it. Instead of expecting complete certainty, families gradually learned to live alongside ongoing medical follow-up while maintaining hope for their children’s future.

### 3.6. Theme 4. Navigating the Healthcare System

Interacting with the healthcare system emerged as a multifaceted continuum for parents, who continuously sought specialised expertise, trusted partnerships, comprehensible information, and long-term care coordination. This trajectory spanned beyond direct medical treatment to include navigating referral channels, communicating across multidisciplinary teams, preparing for post-discharge home care, and managing disjointed services across diverse healthcare settings. Notably, despite expressing substantial confidence in tertiary specialist cardiac centres, families identified systemic gaps in communication, care continuity, and developmental monitoring. These dynamics are further delineated into four interconnected subthemes (Table 4).

#### 3.6.1. Building Trust Through Communication

Parents consistently described communication with healthcare professionals as the foundation of their confidence throughout the disease trajectory. Experiences varied considerably between institutions and individual clinicians; however, clear explanations, empathy, and sufficient consultation time strengthened trust and reduced emotional distress.

Many parents recalled that specialists working in tertiary referral centres carefully explained diagnoses, treatment options, surgical risks, and possible complications. This transparent communication helped families adapt to an otherwise unfamiliar and frightening process.

“*The doctors explained everything in great detail. Because I trusted them, I never lost hope.*”

(P30, Mother, Congenital HD [TGA])

Conversely, communication problems often intensified anxiety. Parents described feeling overwhelmed when medical terminology was used without adequate explanation or when information was inconsistent between healthcare providers. Several also reported that insufficient communication during emergency admissions, intensive care, or the perioperative period increased feelings of uncertainty and helplessness. Rather than requesting more information alone, parents emphasized the importance of receiving information that was timely, understandable, and responsive to their individual concerns.

#### 3.6.2. Preparing Families for Treatment and Home Care

Parents viewed education as an essential component of care throughout diagnosis, surgery, discharge, and long-term follow-up. Although some families felt well-prepared for postoperative care, others described uncertainty regarding medication management, nutritional support, physical activity, wound care, and the use of medical devices after discharge.

Several participants explained that they had to search independently for information on feeding practices, developmental support, medication adjustments, or rehabilitation because these topics received limited attention during routine follow-up.

“*Nobody explained what my child should eat or how to support development after surgery. I had to find that information myself.*”

(P17, Mother, Congenital HD [TOF])

Parents also highlighted that chronic disease management extended beyond cardiac follow-up. They expected routine assessment of growth, nutrition, developmental progress, and psychosocial well-being alongside medical surveillance.

#### 3.6.3. Continuity of Care Beyond Specialist Centres

While specialist cardiac centres were viewed as providing high-quality care, families described considerable challenges in maintaining continuity once they returned to their local communities. Long travel distances, repeated referrals, fragmented follow-up, and differences in clinical experience between healthcare facilities complicated long-term management.

Parents particularly valued coordinated care that reduced unnecessary appointments, minimized travel, and facilitated communication between tertiary centres and local healthcare providers. Families travelling from other provinces emphasized the importance of completing multiple investigations during a single hospital visit whenever possible.

Several parents also expressed the need for an accessible healthcare professional who could coordinate care across specialties and provide ongoing guidance throughout the disease trajectory.

*“Children with chronic diseases should have a healthcare consultant whom families can contact whenever questions arise.*”

(P37, Mother, Acquired HD [KD])

This need reflected families’ desire for continuity rather than simply increased healthcare utilization.

#### 3.6.4. System-Level Needs and Opportunities for Improvement

Parents identified several opportunities to strengthen family-centred cardiac care. Recommendations consistently extended beyond medical treatment itself and focused on improving healthcare organization, coordination, and accessibility. Priority areas included earlier diagnosis, more coordinated referral pathways, integrated nutritional and developmental follow-up, improved psychosocial support, simplified appointment systems for families travelling long distances, and greater availability of specialised laboratory and follow-up services outside major tertiary centres. Across interviews, parents emphasized that comprehensive cardiac care should address not only children’s physiological needs but also their developmental, emotional, and social well-being. These findings suggest that families conceptualized high-quality healthcare as an integrated system capable of supporting both medical management and long-term family adaptation.

### 3.7. Theme 5. Active Family Coping Mechanisms and Behavioural Resource Mobilization

Distinct from their internal emotional trajectory, families actively engaged in practical, behavioural coping mechanisms and mobilized tangible resources to manage daily disease demands. Living with childhood HD required families to continuously adapt to changing clinical circumstances while maintaining everyday family functioning. Parents described coping not as a single strategy but as a dynamic process involving role reorganization, active information seeking, social support, spiritual beliefs, and the gradual development of confidence in managing their child’s condition. As families gained experience, many transitioned from feeling overwhelmed by uncertainty to becoming knowledgeable and active partners in their child’s care. Four interconnected subthemes were identified (Table 4).

#### 3.7.1. Redefining Family Roles and Daily Operations

The diagnosis of HD reshaped responsibilities within the family. Within this predominantly maternal sample, several mothers described taking primary responsibility for coordinating daily care, including medication management, hospital visits, nutritional monitoring, and communication with healthcare professionals. Some of the fathers interviewed described contributing through emotional support, financial responsibilities, practical caregiving, and maintaining opportunities for play and social interaction. These accounts illustrate variation in caregiving roles within the sample rather than systematic differences between mothers and fathers. Families also described adapting routines around the child’s healthcare needs. Employment decisions, household organization, and attention given to siblings were often modified to accommodate repeated hospitalisations, medical appointments, and recovery periods. Rather than perceiving these changes as temporary disruptions, many parents described them as a new family routine that gradually became integrated into everyday life.

#### 3.7.2. Becoming Expert Caregivers

As the disease progressed, many parents reported actively expanding their knowledge beyond information provided during routine clinical consultations. Families searched scientific websites, international medical resources, patient organizations, and online educational materials to better understand their child’s condition. Some described gradually developing sufficient knowledge to participate confidently in clinical decision-making and daily disease management. Several parents explained that they combined professional advice with their own observations to individualize care, particularly regarding nutrition, medication management, and symptom monitoring. This gradual accumulation of experience transformed many parents from passive recipients of information into confident caregivers capable of managing complex medical situations. However, increased information seeking also carried challenges. Parents frequently encountered contradictory advice, misinformation, and overwhelming amounts of online content, sometimes increasing rather than reducing anxiety.

#### 3.7.3. Drawing on Social and Spiritual Resources

Parents relied on multiple sources of support throughout the disease trajectory. Emotional support from spouses, extended family members, and healthcare professionals strengthened families’ ability to cope with uncertainty, while observing positive outcomes in other children with HD often provided reassurance and hope. Religious beliefs also emerged as an important coping resource. Rather than replacing medical care, faith frequently provided emotional stability and facilitated acceptance of uncertain outcomes. Expressions such as “*Whatever God wills*” reflected an attempt to maintain psychological resilience while continuing to pursue all available treatment options. For many families, coping therefore involved integrating practical problem-solving with emotional, relational, and spiritual resources.

#### 3.7.4. Balancing Protection with Independence

One of the most challenging aspects of long-term adaptation involved balancing the desire to protect the child with the need to support normal development and independence. Parents described constantly negotiating decisions regarding physical activity, schooling, peer relationships, and participation in everyday childhood experiences. Some families adopted highly protective parenting styles because of persistent concerns regarding complications or sudden deterioration. Others intentionally encouraged independence, emphasizing that excessive restrictions could reinforce a “sick child” identity and negatively influence self-confidence and social participation.

“*We never wanted our daughter to feel different from other children. If we constantly treated her as sick, she would begin to believe she was.*”

(P39, Mother, HD [SVT])

Over time, many parents described learning to adjust their level of protection according to their child’s clinical stability rather than their own fears. This gradual shift represented an important milestone in family adaptation, reflecting increasing confidence in both the child’s health and their own caregiving abilities.

### 3.8. Theme 6. Healthcare Professionals’ Perspectives on Improving Family-Centred Care

While Themes 1 through 5 captured the lived experiences of the parent cohort, this final theme was exclusively derived from the accounts of the six healthcare professionals. Healthcare professionals described family-centred care as extending beyond the diagnosis and treatment of cardiac disease to include developmental monitoring, caregiver education, psychosocial support, and coordinated long-term follow-up. Although participants acknowledged the high quality of specialised cardiac care, they emphasized that optimal outcomes required stronger integration between tertiary hospitals, primary healthcare services, rehabilitation, and community-based support systems. Their perspectives complemented parents’ experiences by identifying both clinical priorities and system-level opportunities for improving continuity of care. Four interrelated subthemes were identified (Table 4).

#### 3.8.1. Delivering Holistic Care Beyond the Heart

Healthcare professionals consistently emphasized that successful management of childhood HD should not be evaluated solely by surgical outcomes or cardiac function. Instead, they described care as encompassing children’s physical growth, neurodevelopment, nutritional status, psychological well-being, school participation, and quality of life.

Participants noted that many children survive complex cardiac conditions but continue to experience developmental, behavioural, feeding, or psychosocial difficulties that require ongoing assessment. They therefore advocated for routine developmental surveillance alongside cardiological follow-up.

One clinician summarized this perspective:

“*Treating the heart is only one part of the process. Our responsibility continues as the child grows and develops.*”

(HP3)

Healthcare professionals also highlighted the importance of identifying developmental concerns early, enabling timely referral to rehabilitation and supportive services.

#### 3.8.2. Empowering Families Through Education and Partnership

Professionals viewed parents as essential partners in long-term disease management. Effective care therefore depended not only on medical expertise but also on parents’ ability to understand the child’s condition, recognize warning signs, administer treatment correctly, and make informed daily decisions.

Participants emphasized that education should be individualized, repeated throughout follow-up, and adapted to families’ changing needs rather than being limited to discharge instructions. They also acknowledged that parents often searched for additional information independently and stressed the importance of directing families toward reliable evidence-based resources. Several healthcare professionals observed that informed parents participated more confidently in shared decision-making and demonstrated greater adherence to long-term management plans.

#### 3.8.3. Strengthening Multidisciplinary Coordination

All healthcare professionals emphasized that family-centred cardiac care requires effective collaboration across multiple disciplines. In addition to paediatric cardiologists and cardiovascular surgeons, participants identified nurses, dietitians, developmental paediatricians, psychologists, physiotherapists, speech and language therapists, social workers, and primary healthcare providers as important contributors to comprehensive care.

Participants noted that communication between different healthcare providers was not always optimal, particularly during transitions between tertiary hospitals and local healthcare services. Improved coordination was considered essential for ensuring continuity of follow-up and avoiding fragmented care.

One participant explained:

“*No single professional can meet every need of these children. Family-centred care depends on teamwork.*”

(HP2)

Professionals also highlighted the value of standardized referral pathways and structured communication between institutions.

#### 3.8.4. Addressing System-Level Barriers to Family-Centred Care

Although participants expressed confidence in the quality of specialised cardiac services, they identified several structural challenges that limited comprehensive family-centred care. These included high patient volumes, limited consultation time, unequal access to specialised services across regions, and insufficient integration of developmental, nutritional, and psychosocial follow-up into routine cardiology practice. Healthcare professionals recommended strengthening multidisciplinary outpatient clinics, improving access to developmental support services, expanding caregiver education programmes, and enhancing collaboration between tertiary centres and community-based healthcare providers. Several also emphasized the need for greater institutional recognition of family-centred care as an essential component of chronic disease management rather than an optional supportive service.

Overall, professionals viewed high-quality care as a coordinated, longitudinal process in which medical treatment, family education, psychosocial support, and developmental follow-up are integrated across the continuum of care.

### 3.9. Integrated Findings

Integrating parents’ and healthcare professionals’ perspectives demonstrated that caring for a child with HD is a dynamic, multidimensional process extending far beyond the management of cardiac disease. Rather than representing separate themes, participants described an interconnected care journey in which clinical events, family adaptation, healthcare experiences, and system-level factors continuously influenced one another.

Diagnosis emerged as the principal turning point, initiating a process characterised by uncertainty, emotional distress, and increasing dependence on healthcare professionals. As treatment progressed, parents gradually developed disease-specific knowledge and caregiving competence, while healthcare professionals increasingly assumed the role of long-term partners in family adaptation. This reciprocal process strengthened parental confidence and facilitated active participation in children’s care.

Across interviews, both parents and healthcare professionals consistently defined successful treatment in terms of children’s everyday functioning rather than clinical stability alone. Normal growth, developmental progress, adequate nutrition, physical activity, school participation, and social engagement were viewed as equally important indicators of recovery. At the same time, parents’ psychological adaptation evolved alongside children’s clinical course, with hope and confidence increasing over time despite persistent uncertainty regarding future health.

Effective communication emerged as the central mechanism supporting family-centred care. Trust, empathy, continuity of care, and shared decision-making enabled parents to become confident caregivers, whereas fragmented communication and poor care coordination often increased caregiver burden and uncertainty. Both participant groups emphasized that long-term outcomes depend on multidisciplinary collaboration integrating cardiology with developmental, psychological, nutritional, rehabilitation, and community-based services.

Taken together, these findings support an ecological model of family-centred care in childhood HD, in which children’s long-term health and quality of life arise from dynamic interactions among four interrelated domains: the child’s clinical and developmental status, parental adaptation and caregiving competence, therapeutic partnerships with healthcare professionals, and the organization of healthcare services. This integrated framework provides the basis for multidisciplinary interventions that simultaneously strengthen child development, family resilience, caregiver empowerment, and continuity of care.

### 3.10. Ecological Conceptual Framework

The ecological conceptual framework integrates the findings from parents and healthcare professionals within Bronfenbrenner’s Ecological Systems Theory, illustrating how children’s clinical journey and long-term outcomes are shaped through reciprocal interactions across multiple ecological levels rather than by clinical factors alone. Family-centred care is therefore conceptualized as a dynamic process in which communication, caregiver education, multidisciplinary collaboration, and coordinated services facilitate family adaptation and contribute to children’s long-term developmental and family outcomes.

At the centre of the framework is the child, whose clinical journey extends from diagnosis through treatment, recovery, and long-term follow-up. Within the microsystem, families emphasized successful management extends beyond survival to include normal growth, development, participation in everyday life, and quality of life. At the family level, parental psychological adaptation, caregiving competence, emotional support, and confidence developed alongside children’s clinical recovery. At the healthcare team level (mesosystem), effective communication, caregiver education, continuity of care, and multidisciplinary collaboration emerged as key mechanisms linking families with healthcare services.

At the exosystem level, integrated health services, coordinated referral pathways, and community support influenced families’ ability to navigate long-term care. At the macrosystem level, health policies, equitable access, and healthcare organization shaped the broader context in which family-centred care was delivered.

**Figure 1:**
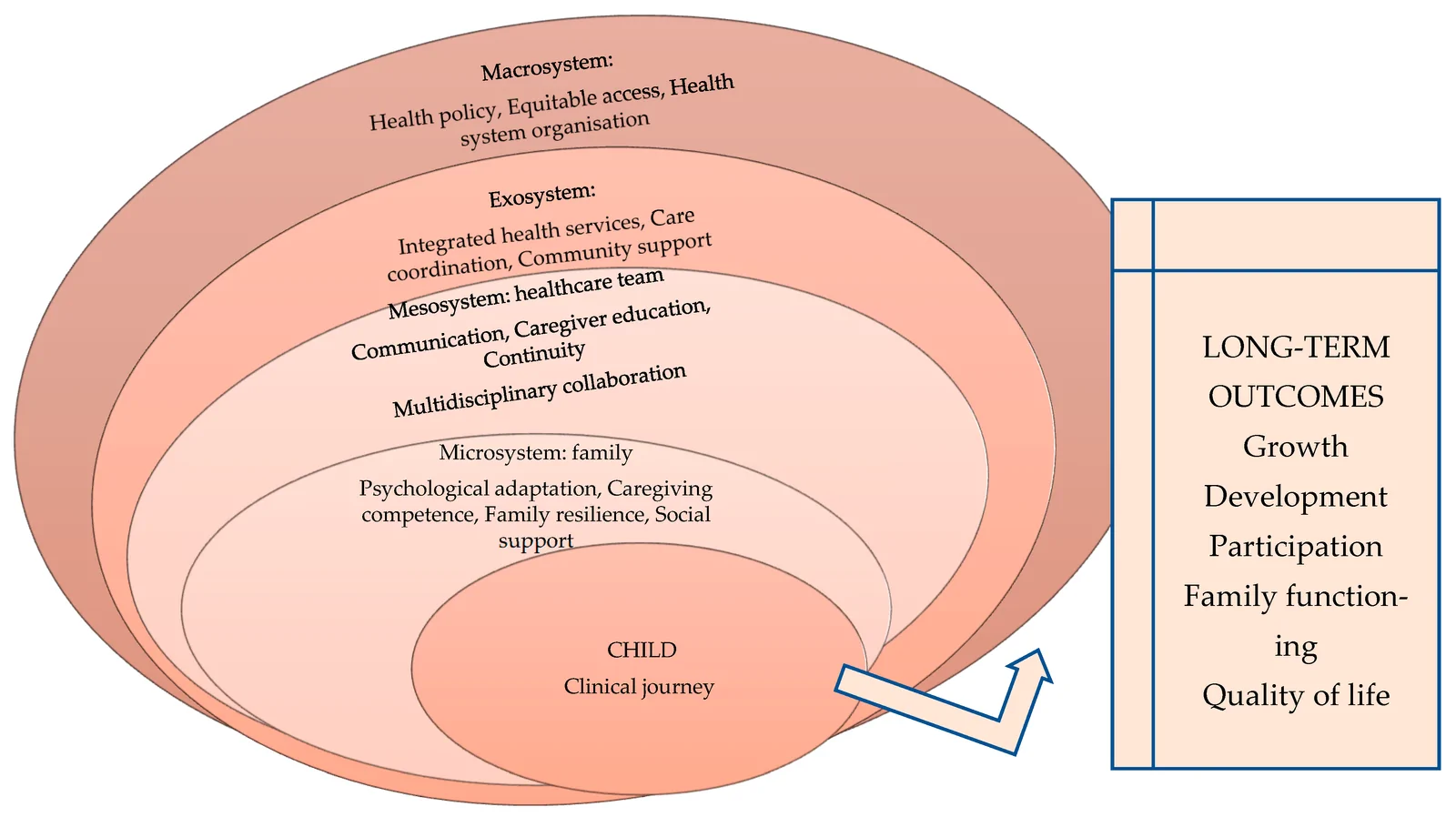
Ecological conceptual framework of family-centred care in childhood heart disease illustrating interactions across Bronfenbrenner’s ecological systems [Clinical journey includes diagnosis, treatment, recovery and long-term follow-up].

Table 5 summarizes the integrated unmet needs and shared priorities identified across these ecological levels. Collectively, the findings indicate that optimal outcomes cannot be achieved through medical treatment alone but require coordinated interventions addressing developmental, psychosocial, educational, organisational, and system-level needs simultaneously. The framework therefore provides a conceptual foundation for designing integrated family-centred models of care for children with HD (Figure 1).

## 4. Discussion

### 4.1. Principal Findings

This study explored the experiences of parents of young children with congenital and acquired HD together with the perspectives of healthcare professionals, providing a comprehensive understanding of family-centred care across the disease trajectory. Rather than depicting childhood HD solely as a cardiac disorder, our findings indicate that it is a long-term developmental and family experience shaped by interactions among the child, caregivers, healthcare professionals, and the wider healthcare system. This interpretation is consistent with ecological and family-centred frameworks, which emphasize that children’s outcomes emerge through dynamic interactions across multiple levels of influence rather than from disease severity alone [11,14,15,18,25,26]. A central contribution of this study lies in showing how clinical, developmental, psychosocial, family, and healthcare-system dimensions interact across the disease trajectory, rather than examining these domains as separate aspects of care. Parents and healthcare professionals identified overlapping priorities, particularly communication, developmental and psychosocial support, multidisciplinary collaboration, and continuity of care; however, they approached these issues from different positions within the care pathway. Parents primarily described their consequences for everyday family life, uncertainty, and caregiving, whereas healthcare professionals more often framed them in terms of service organisation, multidisciplinary resources, referral pathways, and coordination across healthcare settings. Thus, the two perspectives were complementary but not identical, providing insight into both the lived experience of unmet needs and the organisational contexts in which these needs arise.

Overall, our findings support a shift from a disease-centred approach toward an integrated ecological model of family-centred care, in which children’s health and well-being are understood as products of interactions among developmental, psychological, familial, and healthcare system factors. Rather than simply confirming individual challenges previously described in the literature, this perspective brings these dimensions together across the disease trajectory and conceptualises parental adaptation, children’s development, caregiving expertise, and healthcare continuity as interconnected processes. This perspective extends existing qualitative evidence and offers a conceptual basis for reorienting paediatric cardiac services toward integrated, multidisciplinary, and family-centred care.

### 4.2. Beyond Survival: Development, Everyday Functioning, and Participation

Advances in diagnosis, perioperative care, and surgical treatment have transformed childhood HD from a life-threatening condition into a chronic condition with substantially improved survival. As survival has increased, the focus of care has expanded from preventing mortality to optimising children’s long-term development, functioning, and participation. Our findings reinforce this transition by showing that families and healthcare professionals evaluated successful outcomes not only through cardiac recovery but also through children’s neurodevelopment, growth, everyday functioning, participation, and ability to achieve age-appropriate developmental milestones. While contemporary recommendations increasingly recognise developmental outcomes as important indicators of quality in paediatric cardiac care [27,28,29], our findings add a family perspective by showing that parents themselves evaluated recovery through children’s growth, developmental progress, everyday functioning, and participation rather than through cardiac stability alone. The language, speech, and gross motor delays described by parents highlight the vulnerability of young children with HD during critical periods of early brain development. In addition to the physiological effects of congenital heart disease, repeated hospitalisation, prolonged intensive care, and complex surgical interventions may disrupt developmental trajectories. Importantly, the developmental difficulties described in this study cannot necessarily be attributed to HD alone, because repeated hospitalisation, intensive care, surgery, and associated complications may also shape developmental trajectories. Nevertheless, these experiences reinforce the clinical rationale for considering routine developmental surveillance and early intervention as integral components of long-term cardiac follow-up rather than optional adjuncts to specialist care [30,31,32]. Our findings also emphasize that recovery extends beyond physiological stabilization. Improvements in appetite and weight gain following surgery reflected successful metabolic recovery; however, persistent feeding difficulties in some children demonstrate that nutritional challenges may continue after cardiac correction. Together, these findings suggest that clinical and developmental recovery may not proceed in parallel and that developmental and nutritional needs can persist beyond successful cardiac treatment.

This broader understanding of recovery also extended into everyday life. Rather than judging recovery solely by clinical improvement, parents evaluated their children’s health through their ability to participate in ordinary childhood activities, including eating, sleeping, attending school, playing with peers, and engaging in age-appropriate routines. These observations support contemporary models of child health, which recognize participation and everyday functioning as meaningful indicators of successful long-term management in children with chronic conditions [11,33,34,35]. Participation therefore emerged not simply as a consequence of clinical improvement but as an important dimension of long-term recovery itself. Parents’ cautious approaches to physical activity and social participation reflected understandable concerns about fatigue, infection, or future cardiac complications. However, healthcare professionals emphasized that unnecessary restrictions may inadvertently limit children’s physical, social, and emotional development. Similar discrepancies between parental perceptions and clinical recommendations have been reported previously, with overprotective caregiving associated with reduced participation and lower health-related quality of life among children with congenital HD [12,36,37,38]. These findings highlight the importance of individualized guidance that helps families balance appropriate protection with opportunities for normal childhood experiences, independence, and age-appropriate participation.

One novel finding of this study was the widespread use of digital media during prolonged hospitalisation. Parents frequently relied on screens to calm children during procedures, facilitate feeding, or promote sleep, suggesting that hospitalisation itself may unintentionally shape early lifestyle behaviours. Although digital media may provide short-term emotional relief in demanding clinical environments, excessive screen exposure during early childhood has been linked to less favourable cognitive, language, and socio-emotional outcomes [39,40,41,42]. This observation identifies an overlooked aspect of paediatric inpatient care and suggests that developmentally appropriate alternatives, including play-based activities and family interaction, should be incorporated into supportive care programmes.

Overall, these findings support a broader definition of successful paediatric cardiac care in which survival and physiological stabilization are accompanied by attention to development, nutrition, everyday functioning, and participation. Incorporating developmental surveillance, nutritional assessment, guidance on physical activity and healthy routines, and support for age-appropriate participation into routine paediatric cardiology follow-up may better address the evolving needs of children and families throughout the disease trajectory [11,36,39,40].

### 4.3. Parents Undergo a Process of Psychological Adaptation Rather than Experiencing İsolated Emotional Reactions

Our findings suggest that parents’ responses to childhood HD are better understood as a dynamic process of psychological adaptation than as isolated emotional reactions to diagnosis or surgery. Rather than following a linear trajectory, parents described recurring transitions between uncertainty, fear, hope, acceptance, and renewed vigilance as their child’s condition evolved. This pattern is consistent with family adaptation theories, which conceptualize chronic childhood illness as an ongoing process requiring repeated psychological adjustment across different stages of the disease trajectory rather than a single crisis event [11,26,43].

Diagnosis represented the first major disruption to family life, confronting parents with uncertainty regarding prognosis, treatment, and their child’s future. Surgical intervention constituted a second critical transition, simultaneously generating acute anxiety while offering hope for recovery. Similar emotional responses have been widely reported among parents of children with congenital HD and other life-threatening childhood conditions, highlighting that adaptation is shaped by repeated clinical transitions rather than by diagnosis alone [11,43,44,45].

Importantly, psychological adaptation did not parallel clinical recovery. Although successful treatment increased parents’ confidence, persistent vigilance, anticipatory anxiety, and concerns about future deterioration often continued after children became medically stable. This disconnect was also evident in everyday caregiving practices: even when surgery had resolved physiological causes of sleep disturbance, some parents continued co-sleeping or heightened nocturnal monitoring, illustrating how parental vigilance could persist beyond the child’s clinical stabilization [46,47,48]. These observations are consistent with evidence describing prolonged parental distress and paediatric medical traumatic stress following childhood cardiac surgery [49,50,51,52]. They also suggest that clinical stabilization should not be interpreted as an indicator of family recovery.

As parents gained experience, caregiving competence, family support, relationships with healthcare professionals, peer networks, and, for many participants, spiritual beliefs helped restore a sense of control and resilience. However, resilience should not be interpreted as the absence of psychological need. Even highly experienced caregivers frequently described continuing emotional burdens and uncertainty as their children’s clinical and developmental needs changed over time [38,50,52].

These findings highlight the importance of integrating psychosocial care into routine paediatric cardiac services rather than limiting support to families with severe psychological symptoms. Regular assessment of caregiver well-being, anticipatory guidance, and timely access to psychological support at key transition points—including diagnosis, surgery, discharge, and follow-up—may facilitate healthier family adaptation and strengthen both caregiver well-being and children’s long-term developmental outcomes.

### 4.4. Family-Centred Care Depends on Communication Rather than Information Alone

Our findings suggest that effective family-centred care is built on meaningful communication rather than the simple provision of medical information. Although most parents considered the factual information they received about diagnosis and treatment to be adequate, they consistently emphasized that trust, empathy, accessibility, and opportunities for dialogue were equally important in helping them navigate uncertainty. These findings support contemporary models of family-centred care, which conceptualize communication as a collaborative process that fosters partnership, shared decision-making, and caregiver confidence rather than one-way information transfer [11,26]. Communication assumed particular importance during periods of transition, including diagnosis, surgery, hospital discharge, and follow-up. Parents valued healthcare professionals who communicated openly, acknowledged uncertainty when appropriate, and tailored discussions to their emotional and informational needs. This approach appeared to strengthen trust and facilitate adaptation, consistent with previous qualitative studies demonstrating that compassionate and transparent communication enhances family resilience throughout the child’s clinical journey [18,25]. The findings further indicate that communication should extend beyond individual consultations. Discharge was frequently experienced as a period of uncertainty, particularly regarding symptom recognition, medication management, and everyday caregiving.

Medication management represents an important component of the transition from hospital to home. Complex medication regimens and the potential for drug-related adverse effects may add to the monitoring and decision-making demands placed on caregivers of children with HD. Recent evidence among children with CHD has identified polypharmacy and dosing-related factors as contributors to drug-related adverse effects, underscoring the importance of clear caregiver education regarding medication administration, potential adverse effects, and when to seek professional advice [53]. Emerging predictive approaches, including machine-learning-based models, may offer additional opportunities to support medication safety and individualized management; however, their clinical utility and potential contribution to family-centred care require further evaluation [54]. This highlights the importance of staged caregiver education delivered through repeated interactions and reinforced with written or visual resources rather than a single discharge session [18,25]. Similarly, gaps in communication between tertiary centres, primary care, and community services suggest that continuity of care depends not only on clinician–family communication but also on effective coordination across healthcare settings [11,18,25,26].

Taken together, these findings indicate that communication is a core therapeutic process within family-centred care. Sustained partnerships with families, supported by responsive communication, coordinated follow-up, and shared decision-making, may strengthen caregiver confidence and contribute to better developmental, psychosocial, and health outcomes for children with HD.

### 4.5. Families Become Expert Caregivers over Time

Our findings demonstrate that caregiving expertise develops progressively through lived experience rather than being acquired immediately after diagnosis. As families navigated repeated hospitalisations, follow-up visits, and the demands of daily care, they gradually developed the knowledge, practical skills, and confidence needed to manage their children’s condition. This progression reflects experiential learning, whereby caregiving competence evolves through repeated adaptation to changing clinical and developmental challenges. Similar patterns have been described among families of children with congenital HD and other chronic childhood conditions [25,38]. Importantly, this expertise extended beyond technical aspects of disease management. Parents became increasingly confident in recognising symptoms, responding to changes in their child’s condition, supporting nutrition and development, and making informed decisions about when professional care was required. These experiences strengthened parental self-efficacy, a factor consistently associated with improved adherence, more effective caregiving, and better child outcomes [11]. At the same time, families emphasized that caregiving competence developed within supportive relationships involving healthcare professionals, extended family members, peer networks, and, for many participants, spiritual resources. This finding highlights that caregiver expertise is socially constructed rather than achieved through individual effort alone [38,55]. An important implication of these findings is that increasing experience does not eliminate the need for professional support. Even highly competent parents described ongoing uncertainty as children’s needs changed over time, while healthcare professionals emphasized that caregiver learning requires continuous reinforcement rather than one-time education. These observations support a developmental approach to caregiver support in which guidance evolves alongside children’s clinical and developmental trajectories [11,18,26].

Overall, becoming an expert caregiver should be viewed as an outcome of family adaptation rather than an expectation placed on parents. Supporting this process through accessible professional guidance, peer support, and developmentally responsive education may strengthen family resilience and contribute to more effective long-term management of childhood HD.

### 4.6. Healthcare Professionals Advocate a Shift Toward Integrated Family-Centred Care

An important contribution of this study is the inclusion of complementary perspectives from parents and healthcare professionals, which highlight several potential priorities for family-centred paediatric cardiac care. Although healthcare professionals acknowledged the substantial advances in diagnosis, surgical treatment, and survival, they also described developmental and psychosocial needs that may extend beyond these clinical outcomes. Considered alongside parents’ accounts, these perspectives suggest the potential value of an integrated approach that addresses child development, family well-being, and continuity of care alongside biomedical management. This interpretation is consistent with ecological and family-centred models that conceptualise health outcomes as being shaped by dynamic interactions between children, families, healthcare professionals, and healthcare systems [11,26].

Notably, healthcare professionals independently identified many of the same challenges reported by parents, including fragmented services, limited multidisciplinary resources, insufficient psychosocial support, and poor coordination between tertiary centres and community-based services. The consistency of these perspectives strengthens the credibility of the findings and suggests that many unmet needs reflect structural characteristics of healthcare delivery rather than deficiencies in individual clinical practice. Similar organisational challenges have been described internationally, where fragmented care pathways and inadequate coordination remain barriers to comprehensive management of children with chronic conditions [11,18,25,26].

Taken together, these findings indicate that improving outcomes for children with HD requires more than advances in medical treatment alone. The experiences of both parents and healthcare professionals point towards the importance of integrated systems of care in which developmental, nutritional, psychosocial, and medical needs are addressed through coordinated multidisciplinary collaboration across the disease trajectory. In this context, family-centred care emerges not simply as a communication strategy, but as an organising principle for paediatric cardiac services that supports both child development and family adaptation [56].

### 4.7. Implications for Clinical Practice, Health Policy, and Future Research

The findings of this study support a broader approach to long-term follow-up of children with HD that complements cardiac monitoring with developmental surveillance, nutritional assessment, psychosocial support, caregiver education, and anticipatory guidance [57,58]. Structured discharge planning, coordinated referral pathways, multidisciplinary follow-up, and staged education may help families navigate transitions in care and manage continuing uncertainty. For children with CHD, continuity of care ultimately extends beyond paediatric follow-up, as improved survival has created a growing population requiring specialised monitoring into adulthood. Lifelong care therefore requires longitudinal, multidisciplinary assessment that evolves across developmental stages and anticipates the transition from paediatric to adult congenital heart disease services [59]. Although transition to adult care was not directly examined in the present study, the emphasis placed by participants on continuity, evolving care needs, and multidisciplinary follow-up suggests that foundations for such continuity may need to be established during childhood. Recognising parents as active partners in care may further support caregiver confidence and shared decision-making [18,25,60].

At the policy level, family-centred paediatric cardiac care requires accessible and coordinated multidisciplinary services across tertiary and community settings. Strengthening pathways to developmental, nutritional, psychological, rehabilitation, and social support may improve continuity of care and reduce disparities in access to long-term support [56,57,61].

Future research should evaluate family-centred interventions rather than focusing solely on describing experiences. Prospective and longitudinal studies are needed to examine the effectiveness of caregiver education, psychosocial support, developmental follow-up, and coordinated care, and to explore how family adaptation and children’s participation evolve across developmental stages. Studies involving more diverse clinical settings and the direct perspectives of children and adolescents would further extend the present findings. The use of digital media during prolonged hospitalisation also warrants further investigation, particularly in relation to child development and parent–child interaction [18,39,60,62,63].

### 4.8. Strengths and Limitations

This study has several important strengths. The inclusion of both parents and healthcare professionals provided complementary perspectives on family-centred paediatric cardiac care across family, clinical, and healthcare-system contexts. Examining experiences across the disease trajectory also enabled parental adaptation to be understood as an evolving process rather than a series of isolated events. In addition, the ecological framework supported an integrated interpretation of developmental, psychosocial, family, and healthcare-system dimensions.

Several limitations should also be considered. Parents were recruited from a single tertiary paediatric cardiology centre, whereas the six healthcare professionals were recruited from three tertiary centres. The single-centre recruitment of the parent cohort may limit the transferability of parental findings to families receiving care in community-based services or healthcare systems with different organisational structures. In addition, parents were recruited during routine follow-up visits using purposive sampling, which may have introduced selection bias by preferentially including families who remained engaged with specialist follow-up and were available and willing to participate. Conducting parent interviews within the hospital setting may also have influenced participants’ accounts and increased the possibility of socially desirable responses, particularly when discussing their healthcare experiences. Although neither interviewer was involved in participants’ routine clinical care or served as their primary physician, the researchers’ clinical backgrounds may have influenced follow-up questioning, coding, and interpretation. Reflexive field notes, analytical memos, and team discussions were used to make these influences explicit and critically examine them throughout the analytical process. Although healthcare professionals were recruited from three tertiary centres and represented different professional disciplines, the relatively small healthcare professional sample (n = 6) limits the breadth of professional and institutional perspectives captured.

Mothers constituted the majority of parent participants (32 mothers versus 7 fathers), limiting the breadth of paternal perspectives represented and precluding strong conclusions regarding differences between maternal and paternal caregiving experiences. In addition, only six parent participants had children with acquired HD; therefore, the findings should not be interpreted as establishing equivalence between congenital and acquired HD or as supporting condition-specific comparisons. The parent sample was also clinically heterogeneous, with substantial variation in diagnosis, disease severity, surgical history, hospitalisation, complications, and comorbidities. Although this heterogeneity was intentionally incorporated through maximum variation sampling to capture diverse experiences, it limits condition-specific interpretation. Moreover, the effects of HD itself could not always be clearly distinguished from experiences associated with cardiac surgery, prolonged hospitalisation, complications, or coexisting chronic conditions. Recall demands varied across participants because of differences in children’s ages and the timing of diagnosis, treatment, and, where applicable, surgery. Some parents described relatively recent clinical experiences, whereas others reflected on events that had occurred several years earlier. Consequently, accounts of earlier stages of the disease trajectory may have been more susceptible to recall bias than accounts of more recent experiences. Finally, the study explored family experiences through the accounts of parents and healthcare professionals and did not include direct perspectives from children or adolescents with heart disease. Consequently, the findings primarily reflect caregivers’ and professionals’ interpretations of children’s experiences rather than the children’s own voices. Although qualitative research does not aim for statistical generalisability, the contextual detail provided may support assessment of the transferability of the findings to similar tertiary paediatric cardiology settings.

## 5. Conclusions

Based on the accounts of parents and healthcare professionals, this study demonstrates that childhood heart disease, whether congenital or acquired, is experienced not only as a chronic medical condition but also as a long-term developmental and family journey extending well beyond diagnosis, treatment, and survival. Parents described a progressive process of psychological adaptation in which caregiving responsibilities, family roles, and everyday life continually evolved, while healthcare professionals recognised that optimal outcomes depend on addressing children’s developmental, nutritional, psychosocial, and educational needs alongside specialist cardiac management.

The findings further show that family-centred care is sustained through ongoing communication, caregiver empowerment, multidisciplinary collaboration, and continuity across healthcare settings. As parents gradually acquire knowledge, confidence, and caregiving expertise, healthcare systems should recognise and support this evolving role through coordinated developmental surveillance, family education, and accessible psychosocial support.

Overall, the findings suggest that family-centred developmental care represents a common need across childhood heart diseases, regardless of underlying aetiology. Integrating developmental support, family partnership, and coordinated long-term care into routine paediatric cardiac services may improve quality of life, and lifelong well-being while strengthening families’ capacity to manage complex care needs.

## Figures and Tables

**Table 1:** Inclusion and exclusion criteria for participants.

Participant Group	Inclusion Criteria	Exclusion Criteria
Parents	Child aged 0–8 years. Child currently monitored for congenital or acquired HD. Voluntary consent to participate.	Inability to communicate in Turkish. Parent aged under 18 years. Child having a severe acute illness at presentation hindering in-depth interviews/examinations or requiring hospitalisation.
Healthcare Professionals	Active employment in Paediatric Cardiology or Cardiovascular Surgery departments. Clinical experience in the diagnosis, monitoring, or surgical care of paediatric HD patients (as a specialist, surgeon, or nurse). Voluntary consent to participate and be interviewed.	Inability to participate in the interview without disrupting routine unit operations (outpatient clinic, intensive care unit, operating room) due to clinical obligations (patient care, emergencies, or examination schedules).

**Table 2:** Characteristics of the interviewed parents and their children.

	n (%)
Child characteristics	
Gender, male	18 (46)
History of cardiac surgery (yes)	15 (38)
Presence of associated anomalies or comorbidities	8 (21)
Child diagnosis	
Congenital HD	33 (85)
Conotruncal anomalies (TOF: 3; TGA: 3; DORV: 2)	8 (21)
Arrhythmias (SVT: 2; WPW: 1)	3 (8)
Cardiomyopathy, DCM	4 (10)
Semilunar valve disease (BAV:1; VPS:1)	2 (5)
Septal defects (VSD:4; ASD:3)	7 (18)
Others (PAPVD:3; CoA: 2; PDA: 2; Rhabdomyoma:1; TA:1)	9 (23)
Acquired HD (KD: 3; Myocarditis:2; ARF:1)	6 (15)
Characteristics of the interviewed parents	
Parent interviewed, n (%)	
Mother	32 (82)
Father	7 (18)
Age (years), mean ± SD [min–max]	30 ± 6.0 [20–44]
Province of residence, n (%)	
Ankara	17 (44)
Other	22 (56)
Educational level, n (%)	
Primary/secondary education	15 (38)
High school	10 (26)
University degree or higher	14 (36)
Occupation, n (%)	
Civil servant/public employee	13 (33)
Private sector employee/worker	4 (10)
Self-employed/tradesperson	1 (3)
Homemaker	21 (54)
Parental consanguinity	5 (13)
Family history of HD	10 (26)

ARF: Acute Rheumatic Fever (ARF); ASD: Atrial Septal Defect; BAV: Bicuspid Aortic Valve; CoA: Coarctation of the Aorta; DCM: Dilated Cardiomyopathy; DORV: Double Outlet Right Ventricle; HD: Heart Disease; KD: Kawasaki Disease; PAPVD: Partial Anomalous Pulmonary Venous Drainage; PDA: Patent Ductus Arteriosus; SVT: Supraventricular Tachycardia; TA: Tricuspid atresia; TGA: Transposition of the Great Arteries; TOF: Tetralogy of Fallot; VPS: Valvular Pulmonary Stenosis; VSD: Ventricular Septal Defect; WPW: Wolff–Parkinson–White Syndrome.

**Table 3:** Characteristics of healthcare professionals.

Participant Code	Age (Year)	Gender	Profession/Position, Duration of Practice (Years)	Institution
HP1	60	Female	Paediatric Cardiologist (Specialist), 36	Foundation University (Private) Hospital
HP2	46	Female	Paediatric Cardiology Fellow, Physician, 14	Training and Research Hospital
HP3	40	Male	Cardiovascular Surgeon (Specialist), 17	University Hospital
HP4	26	Male	Cardiovascular Surgery Research Assistant Doctor, 3	University Hospital
HP5	52	Female	Cardiovascular Surgery Intensive Care Unit Nurse, 30	University Hospital
HP6	36	Female	Paediatric Cardiology Outpatient Clinic Nurse, 15	University Hospital

**Table 4:** Summary of themes and subthemes identified from interviews with parents and healthcare professionals with quotations supporting each subtheme.

Theme	Subtheme	Illustrative Quotation
1. Living Through the Clinical Journey	Receiving the diagnosis	“…Only after several hospital visits…”
	Navigating treatment and surgical care	“…Surgery gave us hope again…”
	Growth, nutrition and developmental trajectories	“…My baby actually grew very well…”
	Recovery beyond survival	“…Now she can finally play…”
2. Living with HD in Everyday Life	Establishing daily routines around a chronic condition	“…Medication became part of our morning routine…”
	Nutrition, sleep and physical activity as indicators of well-being	“…Doctors encouraged exercise…”
	Preventive health practices and environmental adaptations	“…We became much more careful about infections…”
	Participation in everyday childhood	“…We tried to let him live like every other child…”
3. Parents’ Psychological Responses and Emotional Processing Across the Disease Trajectory	Confronting the diagnosis, Shock	“…My whole world collapsed…”
	Living with uncertainty	“…I still wake up to check her breathing…”
	Rebuilding hope following treatment	“…After surgery we could finally breathe again…”
	Emotional Strains of Parenting and the Normality Dilemma	“…Everything changed after the diagnosis…”
	Looking toward an uncertain future	“…The uncertainty is the hardest part…”
4. Navigating the Healthcare System	Building trust through communication	“…The doctors explained everything…”
	Preparing families for treatment and home care	“…No one told us what to do after discharge…”
	Continuity of care beyond specialist centres	“…We needed someone to coordinate everything…”
	System-level needs and opportunities for improvement	“…Families need one contact person…”
5. Active Family Coping Mechanisms and Behavioural Resource Mobilization	Redefining family roles and Daily Operations	“…My husband supported us while I managed the care…”
	Becoming expert caregivers	“…Eventually we learned everything ourselves…”
	Drawing on social and spiritual resources	“…Faith helped us continue…”
	Balancing protection with independence	“…We tried not to raise a sick child…”
6. Healthcare Professionals’ Perspectives on Improving Family-Centred Care	Delivering holistic care beyond the heart	“…Treating the heart is only one part…”
	Empowering families through education and partnership	“…Families should become partners…”
	Strengthening multidisciplinary coordination	“…No single professional can do this alone…”
	Addressing system-level barriers to family-centred care	“…Coordination between services must improve…”

**Table 5:** Integrated unmet needs, shared priorities, and implications for family-centred care across ecological levels.

Ecological Level	Needs Identified by Parents	Needs Identified by Healthcare Professionals	Key Unmet Needs	Shared Priority	Potential Implications for Family-Centred Care
Child	Normal growth, development, participation in everyday life	Holistic follow-up beyond cardiac status	Comprehensive developmental follow-up	Development-centred cardiac care	Integrate developmental, nutritional, psychological, and rehabilitation services into routine cardiology follow-up
Family	Reliable information, emotional support, confidence in caregiving	Parent education and active partnership	Continuous education, psychosocial support, and caregiver empowerment	Caregiver empowerment	Provide structured education, psychological counselling, peer support, and shared decision-making throughout the disease trajectory
Healthcare team	Trust, communication, coordinated follow-up	Multidisciplinary collaboration	Multidisciplinary and coordinated care	Integrated family-centred care	Establish multidisciplinary family-centred clinics with standardized follow-up pathways and coordinated discharge planning
Health system	Easier access, continuity, coordinated referrals	Better organization and stronger care networks	Accessible, integrated, and equitable long-term care	Longitudinal coordinated care	Strengthen integrated care networks, improve referral systems, expand community-based services, and promote continuity of care across healthcare settings

## Data Availability

The raw data supporting the conclusions of this article will be made available by the authors on request.

## Supplementary Materials

The following supporting information can be downloaded at: https://www.mdpi.com/article/10.3390/healthcare14183018/s1, Materials S1. Semi-Structured Interview Questions for In-Depth Interviews with Parents; Materials S2. Semi-Structured Interview Questions for In-Depth Interviews with Health Professionals.

## Abbreviations

The following abbreviations are used in this manuscript:

ARF	Acute Rheumatic Fever
ASD	Atrial Septal Defect
BAV	Bicuspid Aortic Valve
COREQ	Consolidated Criteria for Reporting Qualitative Research
CoA	Coarctation of Aorta
DCM	Dilated Cardiomyopathy
DORV	Double Outlet Right Ventricle
HD	Heart Disease
KD	Kawasaki Disease
PAPVD	Partial Anomalous Pulmonary Venous Drainage
PDA	Patent Ductus Arteriosus
SVT	Supraventricular Tachycardia
TA	Tricuspid Atresia
TOF	Tetralogy of Fallot
TGA	Transposition of the Great Arteries
VPS	Valvular Pulmonary Stenosis
VSD	Ventricular Septal Defect
WPW	Wolff–Parkinson–White Syndrome
