# Supplementary material for: Beyond Survival: Parents’ Experiences and Healthcare Professionals’ Perspectives on Family-Centred Care for Children with Congenital and Acquired Heart Disease: A Qualitative Study
Source: Healthcare (Basel). 2026 Sep 15;14(18):3018. doi: 10.3390/healthcare14183018 (PMC13606415; doi:10.3390/healthcare14183018)
Supplement: Supplementary file 1 [file healthcare-14-03018-s001.zip › healthcare-4480548-supplementary.pdf]

SUPPLEMENTARY MATERIAL S1

Semi-Structured Interview Questions for In-Depth Interviews with Parents

1. Please describe your child's breastfeeding duration and the transition to complementary feeding following birth.
2. How do you perceive the relationship between your child's eating habits and their underlying heart disease?
3. How would you evaluate your child's growth and development compared with their peers?
4. What activities do you engage in to support your child's development (e.g., reading books, listening to music, playing games)?
5. What is your child's daily physical activity level like, and how has it progressed throughout this process?
6. Could you provide information regarding your child's daily screen time? Which devices does he/she use, when did this usage begin, and what programs does he/she watch?
7. How would you describe your child's sleep routine? Where and at what specific times does he/she sleep, how does he/she initiate sleep, and how many consecutive hours does he/she sleep at night? In what ways were his/her sleep patterns affected during the course of his/her illness?
8. How is your child's daily mobility and physical activity, and how has it been affected during the course of their treatment?Could you you tell us about your child's vaccination status?
9. Who takes care of your child, how have your parental roles been shaped?
10. How did you feel when your child was diagnosed with heart disease and how did the heart disease change your life?
11. What have you done in terms of information, research and support about your child's disease and what are your expectations from the health system?

SUPPLEMENTARY MATERIAL S2

Semi-Structured Interview Questions for In-Depth Interviews with Health Professionals

1. What constitute the most significant phases in the diagnostic and follow-up trajectories of children with congenital and acquired heart diseases?
2. In what ways do these conditions impact the physical growth and neurodevelopmental trajectories of children?
3. What are the most prevalent challenges faced in the management of these pediatric conditions?
4. Regarding which specific subjects do families require information and support most urgently during the diagnosis and treatment phases?
5. What is your assessment of the level of multidisciplinary collaboration (surgeons, cardiologists, nurses, physiotherapists, dietitians, etc.) in the care management of children with heart disease?
6. To what extent do you think families are prepared for the surgical process and involved in the care process? What could be done to increase their participation?
7. To what extent does the healthcare system support the long-term follow-up of children and families' access to healthcare services? What are the main barriers they encounter?
8. Within the current healthcare system, what unmet needs remain for children with heart disease, and which aspects of long-term follow-up are most frequently overlooked?
9. In your opinion, in which areas and in what ways should healthcare professionals working in this field be supported to improve the quality of care?
